# Pollen limitation in the endangered Chinese endemic species *Sinocalycanthus chinensis*

**DOI:** 10.1002/ece3.6550

**Published:** 2020-07-16

**Authors:** Junmin Li, Jingjing Gu, Xinglong Wang, Wenbiao Zhang, Zexin Jin

**Affiliations:** ^1^ Zhejiang Provincial Key Laboratory of Plant Evolutionary Ecology and Conservation Taizhou University Taizhou China; ^2^ Institute of Ecology Taizhou Univesity Taizhou China; ^3^ Xianju Branch of Ecological Environment Bureau of Taizhou City Taizhou China; ^4^ Zhejiang Hangkang Testing Technology Co. Ltd. Hangzhou China; ^5^ Plant Quarantine Station of Beilun District Ningbo City China

**Keywords:** inbreeding depression, outbreeding depression, outcrossing distance, pollen supplementation, selfing, *Sinocalycanthus chinensis*

## Abstract

Pollen limitation negatively impacts endangered and endemic plants with small fragmented populations, such as *Sinocalycanthus chinensis,* an endangered plant endemic to China. In this study, we analyzed the pollen limitation of the *S. chinensis* Damingshan (DMS) population in 2006, 2009, and 2010, and crossed plants with mates separated by different distances, both within and between populations. The DMS population exhibited strong pollen limitation in fruit set, seed set, and seeds per fruit in 2006, 2009, and 2010. The average accumulated pollen limitation (for fruit set times seeds per fruit) was 0.510 ± 0.180. Progeny crossed with pollen from intermediate neighboring plants within the same population (separated by 30–50 m from pollen recipients) had the lowest fitness. No optimal outcrossing distance was found within the DMS population. Progeny from crosses with the Shunxiwu (SXW) and Daleishan (DLS) populations performed relatively better, while those from crosses with Qingliangfeng (QLF) and Longxushan (LXS) populations performed worse. Compared with average reproductive success, outbreeding depression was found in progeny from crosses with the LXS and QLF populations. Reproductive success from pure self‐pollination indicated *S. chinensis* is self‐compatible. Geitonogamous selfing increased reproductive success. Based on geitonogamous selfing, the proportion of selfed offspring was relatively high. These results provide basic references for the conservation of this species.

## Graphical abstract

*Sinocalycanthus chinensis* exhibited strong pollen limitation. No intermediate optimal outcrossing distance was found. Geitonogamous selfing increased the reproductive success.

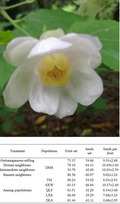

## 1 INTRODUCTION

Pollen limitation, known as the decrease in potential plant reproduction owing to inadequate pollination, is ubiquitous across angiosperms (Ashman et al., 2004; Knight et al., 2005; Larson & Barrett, 2000). Most endangered plant species exist as small and isolated populations, and even natural populations of widespread species are becoming increasingly fragmented (Lázaro & Traveset, 2006). Pollen limitation is especially harmful to endangered and endemic plants with small fragmented populations in which pollination is disrupted (Aizen & Feinsinger, 1994; Fernández, Bosch, Nieto‐Ariza, & Gómez, 2012; Hill, Brody, & Tedesco, 2008).

Pollen limitation may be caused by a decrease in pollen quantity or quality (Aizen & Harder, 2007; Ashman et al., 2004). Pollen quantity limitation occurs when an insufficient quantity of pollen is deposited on the stigma by scarce or ineffective floral visitors (Aloso et al., 2013; Gómez, Abdelaziz, Lorite, Muñoz‐Pajares, & Perfectti, 2010). Pollen quality limitation occurs when pollinators deposit incompatible pollen, self‐pollen, or pollen from closely related individuals (Aloso et al., 2013; Fernández et al., 2012). The occurrence of pollen quantity limitation and quality limitation vary among species and populations and can be affected by the pollinator assemblage, plant population size and density, and other habitat variables (Fernández et al., 2012). The endangered plant *Ottelia acuminate* (Hydrocharitaceae) has been shown to suffer severe pollen limitation owing to a low pollinator visiting frequency (Xia et al., 2013). The endangered plant *Disanthus cercidifolius* Maxim. var. *longipes* H.T. Chang (Hamamelidaceae) was verified to be prone to pollen limitation; however, the pollen source rather than the quantity of pollen had significant effects on the reproduction of this species (Xiao, Zeng, Li, Hu, & He, 2006). Understanding the pollen limitation situation and causes is particularly important for the management and conservation of endangered and endemic plants (Geerts & Pauw, 2012).

In plants, the spatial distance between mates may have negative consequences on progeny fitness in two ways. Crosses over short distances, such as occurs in self‐pollination or crossing between close relatives, may result in inbreeding depression (Charlesworth & Charlesworth, 1987; Oostermeijer, Altenburg, & den Nijs, 1995). In contrast, crosses over long distances may result in outbreeding depression (Price & Waser, 1979; Waser, 1993; Waser & Price, 1994). For most plants, an optimal intermediate outcrossing distance between two mating plants is associated with an optimal degree of outbreeding (Billingham, Simões, Reusch, & Serrão, 2007; Grindeland, 2008; Price & Waser, 1979; Waser, 1993). However, no effect of pollination distance was found to be associated with the reproductive success of several species, including *Hypochaeris radicata* (Becker, Reinhold, & Matthies, 2006) and *Lychnis flos‐cuculi* (Hauser & Loeschcke, 1994). However, few studies have investigated the effects of pollination distance on the reproductive success of endangered species.

*Sinocalycanthus chinensis* Cheng et S. Y. Chang (Calycanthaceae), a tertiary relict species, has been listed as the second most protected plant in China owing to its habitat deterioration and artificial overexploitation (Hu, 2002). *Sinocalycanthus chinensis* is entomophilous (Li & Jin, 2006), and its seeds are enwrapped by pericarp and dispersed only by gravity, which results in the limited dispersal of *S. chinensis* offspring (Li & Jin, 2006). However, there is some potential for self‐compatibility at the late developmental stage, and controlled pollinations studies have shown *S. chinensis* to be self‐compatible and have a mixed mating system (Li, Jin, & Gu, 2012; Zhao et al., 2011). Accordingly, we can hypothesize that pollen limitation, both pollen quality and quantity limitation, might have critical effects in *S. chinensis* populations. However, no empirical data support this hypothesis.

The size of the wild population of *S. chinensis* has contracted, and only a few limitedly distributed populations remain, which have been divided geographically into an eastern group and a western group (Zhang, Chen, Qiu, Li, & Jin, 2001; Zhou & Ye, 2002). Damingshan (DMS) Mountain occurs within the main distribution of the eastern group. In this study, we analyzed the pollen limitation of the *S. chinensis* DMS population from 2006 to 2010 and conducted outcrosses between mates separated by different distance within and among populations. We aimed to characterize the following factors: (a) the intensity of the pollen limitation in *S. chinensis*, (b) the level of inbreeding depression and/or outbreeding depression in the *S*. *chinensis* DMS population, and (c) the optimal outcrossing distance for *S. chinensis*.

## 2 MATERIALS AND METHODS

### 2.1 Plant species

**FIGURE 1:**
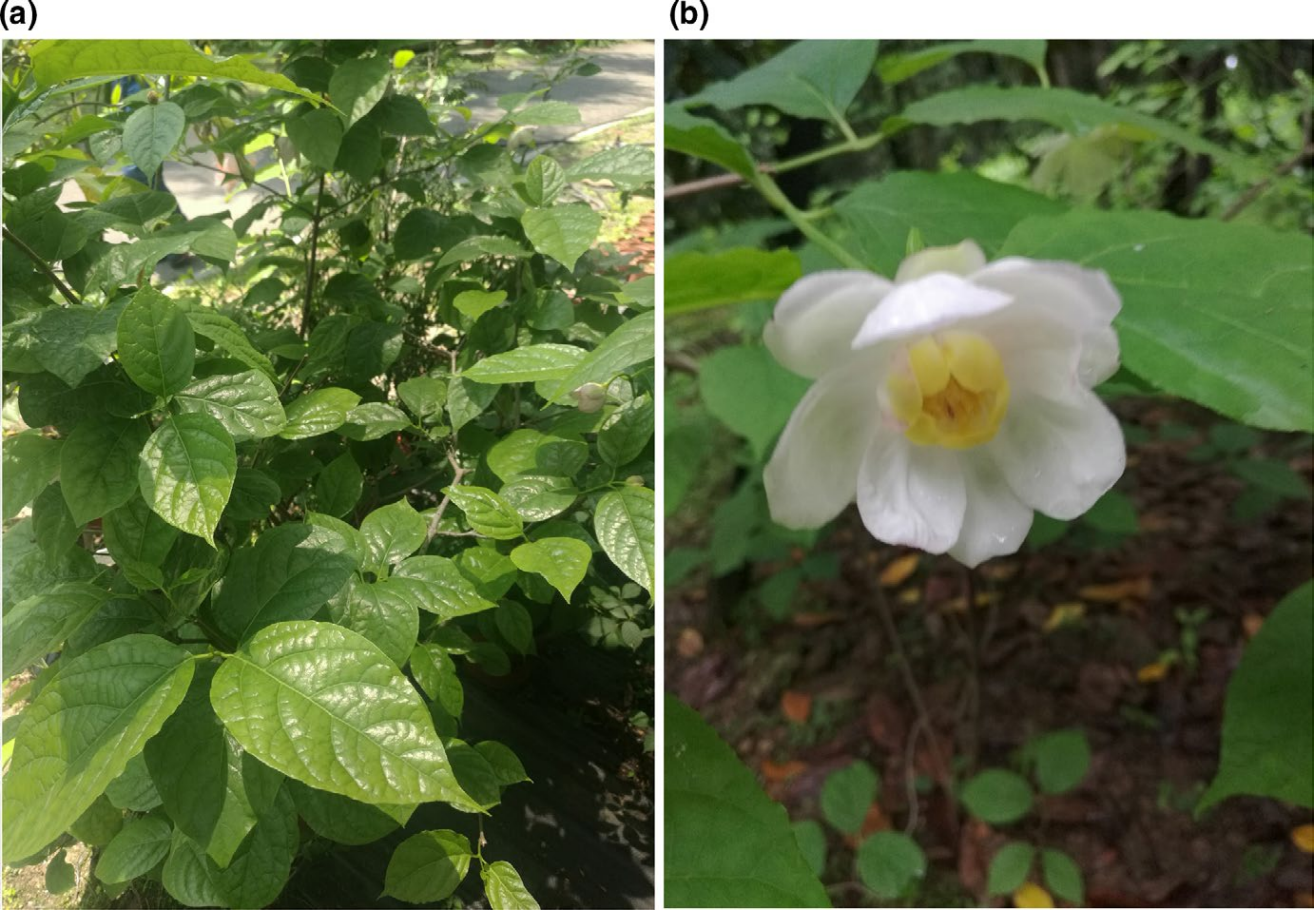
Plant (a) and flower (b) of *Sinocalycanthus chinensis*

*Sinocalycanthus chinensis* Cheng et S. Y. Chang (Calycanthaceae) (Figure 1), an extant representative of its disjunct East Asian‐North American genus, is endemic to Zhejiang Province, China. *Sinocalycanthus chinensis* is a deciduous shrub that grows 1–3 m tall and is scattered under evergreen broad‐leaved forests or mixed evergreen and deciduous broad‐leaved forests around ravines. The distribution spans altitudes from 470 m to 1,200 m (Zhang & Jin, 2009). Previous studies have focused on the morphological anatomy of pollen (Li, 1990), coenology (Zhang et al., 2001; Zhang, Weng, & Xu, 1997), plant chemistry (Ni, Pan, Fu, Wu, & Chen, 2003), reproductive biology (Huang, 1998), and genetic diversity (Li & Jin, 2006; Li et al., 2012; Zhou & Ye, 2002) of *S. chinensis*. *Sinocalycanthus chinensis* is a tertiary relict species, and it is a useful model system for evolutionary and ecological studies in plant biology and genetics.

### 2.2 Study site

This study was conducted at Damingshan Mountain (DMS, 30°02′N, 118°59′E), Linan City, Zhejiang Province, China (Table 1), the location of the largest known extant population (Liu, Zhou, Huang, Bao, & Zhao, 2016). The pollen donor plants were located in the main DMS population, and the other five populations were located at Tashajiang Village (TSJ), Shunxiwu Village (SXW), and Qingliangfeng Mountain (QLF) in Linan City, Zhejiang Province, Daleishan Mountain (DLS) in Tiantai County, Zhejiang Province, and Longxushan Mountain (LXS) in Jixi County, Anhui Province (Table 1). Among these populations, the population size of SXW and QLF was relatively large (Table 1).

**TABLE 1:** Basic characteristics of different *Sinocalycanthus chinensis* populations used in different treatments in this study

No.	Treatments	Population	Longitude	Latitude	Altitude (m)	Slope	Geographical distance to DMS (m)	Habitat	Accompanying species	Number of individuals (#)
1	Geitonogamous‐selfing	DMS	118°59′E	30°02′N	854	NE30°	0	Under an evergreen broad‐leaved forest	*Castanopsis eyrei*, *Daphniphyllum macropodum*, *Nyssa sinensis, Dendropanax dentiger*	≈44,000
2	Immediate neighbours						1–20			
3	Intermediate neighbours						30–50			
4	Remote neighbours						80–100			
5	Among populations	TSJ	119°01′E	30°03′N	928	SE60°	3,712	Under the ever‐green broad‐leaved forest	*Quercus glauca, Platycarya strobilacea, Cyclocarya paliuru, Diospyros glaucifolia*	≈4,000
6		SXW	118°56′E	30°02′N	717	SE55°	4,814	Under the ever‐green broad‐leaved forest	*Cyclobalanopsis glauca*, *Cyclocarya paliurus*, *Acerdavidii Franch, Shima superba*	≈38,000
7		QLF	118°55′E	30°08′N	725	NE50°	12,833	Inside the coniferous and broad‐leaved mixed forest	*Cunninghamia Lanceolata, C. sinensis*, *Platycarya strobilacea*, *Viburnum erosum*	≈36,000
8		LXS	118°42′E	30°04′N	912	NE30°	27,345	Under the ever‐green broad‐leaved forest	*Litsea coreana* var. *sinensis, Symplocos setchuensi, Platycarya strobilacea*	≈6,200
9	Among groups	DLS	120°46′E	28°59′N	782	NE25°	209,128	In the shrub	*Camellia cuspidate, Spiraea salicifolia, Corylopsis sinensis, Rhododendron simsii, Actinidia chinensis, Sargentodoxa cuneata*	≈4,800

### 2.3 Experimental design

#### 2.3.1 Pollen limitation

As a woody shrub, the age class of *S. chinensis* can be estimated from the volume calculated as *d* from the formula $d=\sqrt{a\times b\times c}$, where *a* is the height of the tallest stem, *b* is the crown width along the longitudinal axis, and *c* is the width along the perpendicular axis (Yang, Zhang, Wu, Li, & Zhang, 2006). For *S. chinensis* in the DMS population, the age class ranged from 0 to 3 (Jin, Li, & Liu, 2012). In May 2006, 19 mature plants in age class III (2 < *d *≤ 3) and with similar phenologies that were located in the center of the DMS population and separate from each other by distances of more than 10 m were chosen as pollen recipients. Pollen grains were also collected from 15 pollen donor plants in age class III and with similar phenologies that were located within the same population and separated from each other by a distance of more than 10 m by rubbing a toothpick against newly dehisced anthers. The pollen grains were then mixed in a small plastic vial and stored at 4°C on ice bags in a plastic box, and then the pollen grains were transferred to recipient stigmas within 2 hr (Irwin, 2001). In total, 30 flowers were used for each pollen supplementation, and 30 flowers among 19 naturally open‐pollinated DMS plants were selected as the control (Bossuyt, 2007; Holmes, James, & Hoffmann, 2008). In October 2006, fruits from all tested plants were collected. Fruit set was calculated as the number of mature fruits divided by the number of treatment flowers. Seed set was estimated as the average number of mature seeds divided by the number of fruits within a treatment. (Seeds have a dark, shiny, thickened seed coat when mature. If ovules are not fertilized or aborted, the seeds are very thin, transparent, and whitish in color). The mean number of seeds per fruit was also measured.

For each individual plant, fruit set, seed set, and mean number of seeds per fruit obtained under the control and pollen supplementary treatments were used to calculate the pollen limitation (PL) index as PL = 1 − *C*/*X*, where *C* and *X* represent the fruit set, seed set, and seeds per fruit of the control and cross‐pollination treatments, respectively (Tamura & Kudo, 2000). PL index values can range from 0 (when both treatments produce the same fruit/seed set and/or seeds per fruit, i.e., under no pollen limitation) to 1 (when natural pollination does not produce seeds and hand‐pollination does, i.e., under maximum pollen limitation). A negative PL value means that naturally pollinated flowers received more and/or better pollen than hand‐pollinated flowers (e.g., González‐Varo, Arroyo, & Aparicio, 2009; Lázaro & Traveset, 2006). To estimate the multiplicative pollen limitation throughout fruit set and seeds per fruit, we calculated the cumulative pollen limitation index for each individual as PL_cumulative_ = 1 − (*C*_fruit‐set_ × *C*_seeds per fruit_)/(*X*_fruit‐set_ × *X*_seeds per fruit_) (González‐Varo et al., 2009; González‐Varo & Traveset, 2010).

Similar experiments were conducted in May and October in 2009 and 2010 to obtain two biological replicates. In 2009 and 2010, 100 and 30 flowers for each treatment were used, respectively. We calculated the pollen limitation of the species by averaging PL values over the 3 years.

#### 2.3.2 Optimal outcrossing distance

In May 2009, before *S. chinensis* flowers were open, emasculation and bagging were conducted to protect the stigma, then a pollen supplementation experiment was conducted by pollinating flowers with pollen grains collected from different populations separated by nine different distances (Table 1) to determine the optimal outcrossing distance. Nine treatments were conducted as follows. (a) Geitonogamous selfing was conducted by supplementary hand‐pollination with geitonogamous pollen from the same plant. (b) Immediate neighbor treatments were conducted by supplementary hand‐pollination with xenogamous pollen collected from plants within the same population separated by distances of 1–20 m. (c) Intermediate neighbor treatments were conducted by supplementary hand‐pollination with xenogamous pollen collected from plants within the same population separated by distances of 30–50 m. (d) Remote neighbor treatments were conducted by supplementary hand‐pollination with xenogamous pollen collected from plants within the same population separated by distances of 80–100 m and thus unlikely to interact via open pollination. The within‐population distances from the center of the DMS population were measured with tape measures. (5–9) Between population treatments were conducted by supplementary hand‐pollination with xenogamous pollen collected from plants in the TSJ, SXW, QLF, LXS, and DLS populations, respectively. Geographical distances were calculated using Earth Explorer 4.0, and a detailed summary of the nine treatments is provided in Table 1. Thirty flowers from 19 mature plants in age class III (2 < *d* ≤ 3), with similar phenologies, and that were located in the center of the DMS population and separated from each other by a distance of more than 10 m were chosen as pollen recipients for each treatment. Pollen grains were collected from 15 pollen donor plants in age class III, with similar plant phenologies, separated from each other by a distance of more than 10 m, and located in the different populations in Table 1 by rubbing a toothpick against newly dehisced anthers. The pollen grains were then mixed in a small plastic vial and stored at 4°C on ice bags in a plastic box, and then the pollen grains were transferred immediately to recipient stigmas. According to a previous study by Zhang and Jin (2009), the pollen viability of *S. chinensis* lasts for 5 days after collection (no significant difference was detected among pollen of different days). The within‐population pollen grains were used for hand‐pollination within 2 hr, while the between‐population pollen grains were used for hand‐pollinations within 6 hr, without an observed decrease in the viability of the pollen grains. All hand‐pollinated stigmas were saturated with pollen grains. After treatment, bagging was conducted on the flowers to avoid the contamination of other pollens.

To quantify levels of inbreeding depression and outbreeding depression, we calculated relative performance of crosstypes (*R*_P_) for fruit set, seed set, and seeds per fruit (Weisenberger, 2012). For treatments 2, 3, and 4, inbreeding and outbreeding depression were evaluated as *R*_Pi_ = (*W*_ow_ − *W*_s_)/*W*_max_, where *W*_s_ is the fitness of selfed progeny with geitonogamous pollen grains, *W*_ow_ is the fitness of the progeny from crosses within populations, and *W*_max_ is the maximum value between *W*_ow_ and *W*_s_. *R*_Pi_ values varied from −1 to 1 (Quilichini, Debussche, & Thompson, 2001), where positive *R*_Pi_ values indicate that outcrossed progeny within populations are more fit than selfed progeny, and inbreeding depression is consequently present; negative *R*_Pi_ values indicate outbreeding depression.

For treatments 5–9, heterosis or outbreeding depression was calculated as *R*_Ph_ = (*W*_ob_ − *W*_o_)/*W*_max_ (Weisenberger, 2012), where *W*_ob_ is the fitness of progeny from crosses between populations, *W*_o_ is the fitness of progeny from crosses within populations averaged together, and *W*_max_ is the maximum value between *W*_o_ and *W*_ob_. *R*_Ph_ values range from −1 to 1, where positive *R*_Ph_ values indicate heterosis and progeny from crosses between populations outperform progeny from crosses within populations; negative *R*_Ph_ values indicate outbreeding depression between populations.

The significance of inbreeding depression and outbreeding depression for each treatment was tested using a one‐sample *t* test (test value = 0). The significance of *R*_Pi_ or *R*_Ph_ values between two different treatments was tested by means of a paired *t* test.

#### 2.3.3 Geitonogamous selfing

In May 2009, when *S. chinensis* flowers were open, the following two treatments were conducted. (a) Pure self‐pollination treatments were conducted by enclosing the flowers of *S. chinensis* within paper bags while the flowers were still in bud and then leaving them untouched. (b) Geitonogamous‐selfing treatments were conducted by supplementary hand‐pollination with geitonogamous pollen grains from the same plant. To estimate the mating structure, we calculated the proportion of selfed offspring (*s*) in a natural population as *s* = (*W*_n_ − *W*_o_)/(*W*_s_ − *W*_o_) (Charlesworth, 1988; Fischer & Matthies, 1997), where *W*_n_ indicates the fitness of progeny from naturally open‐pollinated flowers, *W*_o_ indicates the fitness of progeny from geitonogamously pollinated flowers, and *W*_s_ indicated the fitness of progeny from self‐pollinated flowers.

## 3 RESULTS

### 3.1 Pollen limitation

In 2006, pollen addition increased fruit set, seed set, and seeds per fruit by 6.1%, 46.8%, and 26.2%, respectively (Table 2). In 2009, pollen supplementation increased fruit set, seed set, and seeds per fruit by 26.9%, 66.0%, and 40.2%, respectively (Table 2). In 2010, pollen supplementation increased fruit set, seed set, and seeds per fruit by an average of 26.5%, 36.1%, and 40.3%, respectively (Table 2). Paired *t* test results showed that pollen limitation in 2006 was significantly lower than in 2009 (*t *= −4.280, *p* = .023), than those in 2010 (*t *= −6.519, *p* = .007). However, there was no significant difference in pollen limitation indices between 2009 and 2010 (paired *t* test = 1.975, *p* = .143). The accumulated pollen limitation was 0.510 ± 0.180, indicating that pollen limitation was strong in *S. chinensis*. The effect of pollen quantity on fruit set was significantly lower than on seed set (paired *t* test = −8.769, *p* = .013).

**TABLE 2:** Fruit set, seed set, and seeds per fruit produced by naturally open‐pollinated flowers (control), under xenogamous pollen supplementation treatments in *Sinocalycanthus chinensis*, as well as the pollen limitation (PL) index

Variable	Year	Control	Xenogamous pollen supplementary treatment	PL	Averaged PL (mean ± *SD*)
Fruit set	2006	0.6112	0.6506	0.061	0.264 ± 0.204
	2009	0.4211	0.7910	0.468	
	2010	0.5652	0.7662	0.262	
Seed set	2006	0.5913	0.8085	0.269	0.444 ± 0.199
	2009	0.2186	0.6433	0.660	
	2010	0.4248	0.7109	0.402	
Seeds per fruit (#)	2006	7.78	10.5900	0.265	0.343 ± 0.070
	2009	6.70	10.4900	0.361	
	2010	5.69	9.5254	0.403	
Fruit set × seeds per fruit	2006	4.76	6.8899	0.310	0.510 ± 0.180
	2009	2.82	8.2976	0.660	
	2010	3.22	7.2984	0.559	

### 3.2 Optimal outcrossing distance

Fruit set, seed set, seeds per fruit under the xenogamous treatment within populations showed that progeny of crosses with pollen collected from intermediate neighbors within the same population (30–50 m from recipient plants) had the lowest fitness. Based on this xenogamous cross‐result, no inbreeding depression was identified in the geitonogamous selfing treatment (*R*_Pi_ < 0, one‐sample *t* test, *t *= −2.793, *p* = .068). In contrast, based on the other two xenogamous cross‐results (i.e., of the immediate neighbor and remote neighbor treatments), inbreeding depression existed in *S. chinensis*, but was not particularly strong (i.e., *R*_Pi_ was larger than 0). There are significant and marginally significant differences in *R*_Pi_ between the progeny from the immediate neighbor and intermediate neighbor treatments (paired *t* test, *t* = 3.524, *p* = .039) and the remote neighbor and intermediate neighbor treatments (paired *t* test, *t *= −2.534, *p* = .085), respectively (Table 3).

**TABLE 3:** Fruit set, seed set, and seeds per fruit under the nine pollen supplementation treatments and the relative performance of crosstypes (*R*_P_) for fruit set, seed set, and seeds per fruit, and fruit set times seeds per fruit in *Sinocalycanthus chinensis*

No.	Treatment	Population	Fruit set	Seeds set	Seeds per fruit	*R*_P_			
						Fruit set	Seeds set	Seeds per fruit	Fruit set × seeds per fruit
1	Geitonogamous‐selfing	DMS	71.15	54.66	9.91 ± 2.68	–	–	–	–
2	Distant neighbours		79.10	64.33	10.49 ± 3.04	0.1005	0.1503	0.0553	0.1504
3	Intermediate neighbours		54.79	42.60	10.03 ± 2.59	−0.2299	−0.2206	0.0121	−0.2205
4	Remote neighbours		80.56	60.07	9.62 ± 3.24	0.1168	0.0901	−0.0293	0.0903
5	Among populations	TSJ	88.24	54.92	8.03 ± 2.91	0.1899	−0.0135	−0.2010	−0.0131
6		SXW	83.15	66.84	10.37 ± 2.49	0.1403	0.1671	0.0309	0.1673
7		QLF	63.51	32.20	6.54 ± 3.68	−0.1115	−0.4216	−0.3493	−0.4215
8		LXS	66.00	39.29	7.68 ± 3.24	−0.0767	−0.2942	−0.2358	−0.2940
9	Among groups	DLS	81.44	61.11	9.68 ± 2.95	0.1223	0.0890	−0.0368	0.0892

Note

*R*_Pi_ is shown for treatments 2, 3, and 4, while *R*_Ph_ is shown for treatments 5, 6, 7, 8, and 9.

Fruit set, seed set, seeds per fruit under the xenogamous pollen cross‐treatment among populations showed that progeny in treatments crossed with the SXW and DLS populations performed relatively better. Based on these two xenogamous cross‐results, outbreeding depression was not observed in treatments crossed with SXW (*R*_Ph_ > 0, one‐sample *t* test, *t* = 3.894, *p* = .030) and DLS (*R*_Ph_ > 0, one‐sample *t* test, *t* = 9.051, *p* = .012) populations, except for with respect to seeds per fruit (Table 3). Moreover, those comparisons also showed that progeny in treatments crossed with QLF and LXS populations performed relatively worse. Based on the results of these two xenogamous crosses, outbreeding depression was identified in the treatment crosses with QLF (*R*_Ph_ < 0, one‐sample *t* test, *t *= −4.436, *p* = .021) and LXS (*R*_Ph_ < 0, one‐sample *t* test, *t* = 9.051, *p* = .012) populations, except with respect to seeds per fruit (Table 3).

### 3.3 Geitonogamous selfing

Experiments conducted to assess levels of self‐compatibility showed that *S. chinensis* is self‐compatible. Pure self‐pollination crosses yielded 9.09% fruit set, 2.01% seed set, and 2.86 ± 0.86 seeds per fruit. In 2009, compared with naturally open‐pollinated fruit set, seed set, and seeds per fruit, the geitonogamous pollination treatment yielded increases of 68.96%, 150.05%, and 47.91%, respectively; based on the geitonogamous pollination selfing treatment, the proportion of selfed offspring (*s*) according to fruit set, seed set, and seeds per fruit were 0.4679, 0.6229, and 0.4557, respectively. In 2010, supplementary treatment with autogamous pollen grains showed that the fruit set and seed set were 0.5690 and 0.7960, respectively.

## 4 DISCUSSION

In this study, we found the DMS population of the endangered *S. chinensis* exhibited strong pollen limitation, and the pollen supplementation, based on fruit set times seeds per fruit, increased reproductive output by 0.510 ± 0.180 seeds in 2006, 2009, and 2010. Until now, several fragmented endangered plants were also found to suffer high pollen limitation, including *Brunsvigia litoralis* (Geerts & Pauw, 2012), *Dracocephalum austriacum* (Castro, Dostálek, van der Meer, Oostermeijer, & Münzbergová, 2015), *Disanthus cercidifolius* Maxim. var. *longipes* H.T. Chang (Xiao et al., 2006), and *Eremosparton songoricum* (Shi, Wang, Zhang, Gaskin, & Pan, 2010). Pollen limitation has been widely reported in fragmented, isolated, and sparse populations, both for anemophilous and entomophilous taxa (Knapp, Goedde, & Rice, 2001; Lyon, 2010; Rocha & Aguilar, 2001). Recently, the number and size of wild *S. chinensis* populations have decreased with increases in anthropogenic activities (Zhang, 2007). The habitat of *S. chinensis* was gradually fragmented and limited to small isolated areas and eventually was further divided into small, island‐like populations (Li et al., 2012). Pollen limitation is particularly likely in small, isolated habitats (Lázaro & Traveset, 2006; Lyon, 2010), owing to the lower number of potential mates or suitable pollinators (Zhang & Lou, 2015). This scenario matches the situation of the fragmented endangered *S. chinensis*.

Plant reproductive success often depends on pollination, mating systems, and population habitats, among other factors (Aizen & Harder, 2007; Rymer, Whelan, Ayre, Weston, & Russell, 2005). The reproductive success of *S. chinensis* might be affected by the floral structure of *S. chinensis*. Flowers of *S. chinensis* are protogynous with female organs maturing earlier than male organs within the same flower (Zhang & Jin, 2009). However, there is overlap between male and female reproductive stages (Zhang & Jin, 2009; Zhao et al., 2011). Thus, autogamous selfing is possible when pollen from matured stamens falls onto matured stigmas. In this study, we found the fruit set of pure selfing to be 9.09%, indicating the self‐compatibility of *S. chinensis*. In general, self‐compatible plants can be considered facultatively autogamous (Aguilar, Ashworth, Galetto, & Aizen, 2006). Although self‐compatible species usually require animal pollinators to transport pollen from other conspecific individuals, either selfing (autogamous or geitonogamous crosses) or outcrossing (xenogamous crosses) can yield seeds (Aguilar et al., 2006). In addition, *S. chinensis* is entomophilous and pollinated by small insects with limited or no flight, which leads to flowers being visited mainly within the same individual plant by any one pollinator (Zhang & Jin, 2008). Protogynous flower traits can decrease the rate of selfing (Reusch, 2001), which might increase geitonogamous selfing versus autogamous selfing. The limited pollinators in the DMS populations (Zhang & Jin, 2008) and protogyny of flowers are the main factors underlying the strong pollen limitation in *S. chinensis* and why the reproductive success of geitonogamous selfing was high. Similar results have been observed in the endangered protandrous self‐compatible species *Dracocephalum austriacum* (Castro et al., 2015).

In this study, we found that pollen limitation of the *S. chinensis* DMS population varied among years, especially in 2009, as indicated by seeds set and seeds set × seeds per fruit, suggesting that reproductive success of *S. chinensis* is affected by climatic, geographic, and environmental factors in the DMS population across different years. Fernández et al. (2012) found that some environmental variables, such as annual rainfall, were positively associated with pollen limitation, and bare soil cover had a marginally significant negative effect on pollen limitation in *Erysimum popovii*, an endangered narrow endemic crucifer. According to the water resources bulletin of Zhejiang Province, China, the annual mean precipitation totals in the Summers of 2006, 2009, and 2010 were 439.9 mm (https://www.docin.com/p‐185789189.html), 588.0 mm (https://www.docin.com/p‐68743863.html), and 612.1 mm (http://zj.weather.com.cn/qhbh/qhgb/08/1435990.shtml), respectively, while the annual mean temperatures were 18.5°C, 18°C, and 17.5°C, respectively. According to our field survey, compared with 2009 and 2010, the climate in 2006 was mild, and most days were sunny during the flowering period. Based on the high reproductive success of the control, we predicted that the low pollen limitation in 2006 might be owing to the low response of plants to the pollen supplementation experiment (Table 1), which might have contributed to the natural high pollination success growing in years with lower mean precipitation totals and higher mean temperatures. Fernández et al. (2012) suggested that plants growing in areas with low rainfall are unable to respond to supplementary pollination by increasing their seed number. Haig and Westoby (1988) found that in populations with low rainfall, seed production is likely to be limited by water availability or by a combination of water availability and pollen. In addition, the high reproductive success in the control in 2006 could also be owing to more pollen naturally reaching *S. chinensis* stigmas in a year with low mean precipitation. Significant negative correlations between pollen influxes and relative humidity and vapor pressure in summer were found in majority arboreal pollen (Li, 2013). However, the reasons for the lowest reproductive success of the control and the highest pollen limitation occurring in 2009, given the intermediate annual mean precipitation and temperature, remain unknown. Forsyth (2003) found that the reproductive success (percent seed set) was significantly correlated with the number of plants flowering annually, which varied greatly among years. In addition, they found that plants flowering asynchronously were pollen‐limited, whereas plants flowering synchronously were not (Forsyth, 2003). Further research should focus on the relationship between pollen limitation and the number of flowers and flowering synchrony in *S. chinensis*.

Determination of the optimal outcrossing distance provides support for the management of this endangered species. Waser and Price (1994) describe a demographic study of F1 progeny resulting from hand‐pollinations of *Delphinium nelsonii* plants separated by a range of crossing distances and found that inbred and outbred progeny, resulting, respectively, from short‐ and long‐distance crosses, performed more poorly than progeny from crosses over intermediate distances, both in terms of survival and eventual reproduction by flowering. The intermediate optimal outcrossing distances were also found in the monocarpic angiosperm *Ipomopsis aggregata*, with outcrossing distances of 10 m producing more offspring with higher fitness than those separated by outcrossing distances of 1 m or greater than 30 m (Waser, Price, & Shaw, 2000). However, in this study, we did not find such results at intermediate outcrossing distances in the three crossed treatment within the same populations. Similarly, several studies have found no evidence of an optimal outcrossing distance in *Sabatia angularis* (Dudash, 1990), *Yucca whipplei* subsp. *whipplei* (Richter & Weis, 1998), and *Pyrus ussuriensis* var. *ovoidea* (Yin, 2002). This phenomenon might be explained by three factors. First, there existed intermediate outcrosses between 1 m (immediate neighbors) and 50 m (intermediate neighbors) or between 50 m and 100 m (remote neighbors); however, we did not find them among the relatively small number of experimental treatments. By using intersimple sequence repeat (ISSR) markers, Jin et al. (2012) detected a significant spatial genetic structure at a distance less than 20 m and a distance of 90 m in the DMS population of *S. chinensis*. Billingham et al. (2007) suggested that the genetic structuring might result in both inbreeding and outbreeding depression, thus determining an optimal intermediate outcrossing distance. The spatial genetic structure of *S. chinensis* indicated that the intermediate outcross distance would likely be between 20 m and 90 m. Further studies should assess more treatments with distances between 20 m and 90 m to identify optimal outcrossing distances more accurately. Second, pollen limitation might not be the sole or dominant determinant of the reproductive success of *S. chinensis* in the DMS population; other factors, such as postpollination and postfertilization selection, may also affect the reproductive success of *S. chinensis* (Irwin, 2001). Third, inbreeding depression may be cryptic and difficult to assess in species with a long history of selfing (Weller, Sakai, Thai, Tom, & Rankin, 2005). *Sinocalycanthus chinensis* is self‐compatible, with a mixed mating system. The long history of selfing of S*. chinensis* might weaken the effect of outcrossing distances on the reproductive success of this species.

Outbreeding depression is the population‐level counterpart to mechanisms separating species or subspecies (Schierup & Christiansen, 1996). Progeny from crosses with the DLS population performed relatively better, and outbreeding depression was absent in treatments crossed with the DLS population. Zhao, Zhou, Liu, and Bao (2014) also found that outbreeding with the DLS population was apparently more dominant than that with the other treatments. This ecological mechanism assumes that subpopulations are differentiated by adaptation to different environments and that crosses between sites are then expected to yield maladapted offspring, resulting in outbreeding depression (Schierup & Christiansen, 1996). There is high genetic differentiation between the DLS and DMS populations (Li et al., 2012). The heterosis produced by the increased interpopulational gene flow between the DMS and DLS populations might diminish any outcrossing depression. However, in this study, progeny in treatment crosses with QLF and LXS populations performed relatively worse and outbreeding depression existed in the treatment crosses with the QLF and LXS populations. The observed outbreeding depression might be owing to the lower pollen quality of the QLF and LXS populations. The LXS and QLF populations are located on the dark foothills in the northern faces of mountains, and the plants are located under the canopies of evergreen broad‐leaved forests. Both of the populations were small, and only a few individuals were found in each population. The shady habitat and the lower diversity of the populations may weaken the pollen quality, resulting in low reproductive success, that is, observed outbreeding depression in the context of this study. In addition, we found that progeny in the treatment crosses with the SXW and DLS populations performed relatively better, and no outbreeding depression was found compared with the average reproductive success in treatments crossed within the same population. SXW and DLS populations are located along the southern faces of mountains, and their sunny habitats may thus increase the pollen quality. Somewhat surprisingly, outbreeding depression for fruit set and seed set can be detected but not for seeds per fruits, indicating that the observed outbreeding depression often occurs in the number of ovules but not in ovule fertilization. The inflorescences of *S. chinensis* are showy single flowers with high ornamental value but without a fragrance. As a single flower, the number of pollen grains deposited on the stigmas of *S. chinensis* might be an important factor restricting fruit (i.e., seed) production.

In conclusion, *S. chinensis* exhibited strong pollen limitation. No intermediate optimal outcrossing distance was found. Inbreeding depression was found for the intermediate neighbor crosses within the same population (30–50 m). Outbreeding depression was found in treatment crosses with the LXS and QLF populations. Geitonogamous selfing increased the reproductive success. Nevertheless, there were many factors potentially affecting the results, such as the unknown level of pollen manipulation and the possible quality differences between supplementation and control treatments (Ashman et al., 2004), and thus, caution should be applied in interpreting these results. In addition, to more accurately assess the potential optimal outcrossing distance in *S*. *chinensis*, future research should increase the number of experimental populations separated by different distances, the number of populations in total, and the range of distances within individual populations.

## CONFLICT OF INTEREST

None declared.

## AUTHOR CONTRIBUTIONS

**Junmin Li:** Conceptualization (equal); formal analysis (equal); writing–original draft (lead); writing–review and editing (lead). **Jingjing Gu:** Data curation (equal); formal analysis (equal); investigation (equal); methodology (equal). **Xinglong Wang:** Data curation (equal); formal analysis (equal); investigation (equal); methodology (equal). **Wenbiao Zhang:** Data curation (equal); formal analysis (equal); investigation (equal); methodology (equal). **Zexin Jin:** Conceptualization (lead); funding acquisition (lead); project administration (lead); resources (lead); supervision (lead); validation (lead); writing–original draft (equal); writing–review and editing (equal).

## Data Availability

The data were deposited in Dryad (https://doi.org/10.5061/dryad.j6q573n9t).
